# Quantitative Videofluoroscopic Analysis of Postoperative Swallowing Outcomes in Patients with Oral and Oropharyngeal Cancer 

**DOI:** 10.22038/ijorl.2025.90978.4039

**Published:** 2026

**Authors:** Quang Xuan Ly, Duc Tan Vo, Chau Minh Le Tran, Loan Thi Hong Nguyen

**Affiliations:** 1 *Department of Otolaryngology – Head and Neck Surgery, University Medical Center Ho Chi Minh City, Vietnam.*; 2 *Department of Radiology, University Medical Center Ho Chi Minh City, Vietnam.*; 3 *Department of Radiology, School of Medicine, University of Medicine and Pharmacy at Ho Chi Minh City, Vietnam.*; 4 *Department of Otolaryngology – Head and Neck Surgery, School of Medicine, University of Medicine and Pharmacy at Ho Chi Minh City, Vietnam.*

**Keywords:** Dysphagia, Oral Cancer, Oropharyngeal Cancer, Videofluoroscopic Swallowing Study (VFSS), Swallowing Biomechanics

## Abstract

**Introduction:**

Postoperative dysphagia significantly affects the quality of life of patients with oral and oropharyngeal cancer. We aimed to objectively analyze swallowing function in these patients using quantitative biomechanical indices from Videofluoroscopic swallowing studies (VFSS) as well as to identify independent predictors of key swallowing outcomes.

**Materials and Methods:**

We included 60 patients with postoperative oral and oropharyngeal cancers. VFSS were performed at 3 months (for patients without adjuvant therapy) or 6–7 months (for patients with adjuvant therapy) to assess swallowing safety (Penetration-Aspiration Scale); post-swallowing pharyngeal residue; and biomechanical functions, including pharyngeal transit time (PTT), laryngeal vestibule closure duration, pharyngoesophageal segment opening duration/dimension, and laryngeal elevation. Generalized linear models (GLMs) were used to identify independent predictors.

**Results:**

The cohort was predominantly male (78.3%); further, 58.4% of the patients received adjuvant therapy. Thin liquids had the highest penetration and aspiration rates, whereas extremely thick liquids had the lowest rates. The GLM identified sex, adjuvant treatment, and suprahyoid muscle defects as independent predictors of different VFSS parameters. Specifically, adjuvant therapy and sex were associated with a prolonged PTT, while suprahyoid muscle defects were significant predictors of reduced laryngeal elevation.

**Conclusion:**

Swallowing impairment remains prevalent after surgery for oral and oropharyngeal cancers. VFSS analysis could highlight specific biomechanical deficits; further, we identified predictors of key swallowing outcomes, including sex, adjuvant treatment, and suprahyoid defects.

## Introduction

Dysphagia or swallowing impairment is a significant and prevalent morbidity among patients undergoing treatment for oral cavity and oropharyngeal cancer (1-3). These treatments, which usually involve extensive surgical resection, radiotherapy, or a combination of therapies, can severely compromise swallowing function and profoundly affect the patient's quality of life (1,4,5). Specifically, surgical interventions, especially extensive resection of the tongue and base of the tongue (BOT), are significantly associated with postoperative swallowing function (6). Damage to or resection of the suprahyoid muscles, including the geniohyoid and mylohyoid muscles, is especially critical. This can be attributed to the crucial role of these muscles in hyoid bone movement, airway protection, and opening of the pharyngoesophageal segment (PES), resulting in severe postoperative swallowing dysfunction (7). The tumor extent is associated with swallowing outcomes and aspiration risk, with higher tumor stages and larger resections being strong risk factors for postoperative swallowing dysfunction (8). Furthermore, post-swallowing pharyngeal residue is associated with impaired tongue base movement, laryngeal movement, and cricopharyngeal muscle function, which indicates issues with bolus propulsion and clearance (9). Additionally, adjuvant treatments, including radiotherapy and chemoradiotherapy, are associated with severe and persistent swallowing impairments (10). These treatments induce tissue damage, including mucositis, xerostomia, trismus, and fibrosis, which are associated with reduced range of motion in pharyngeal structures and an increased aspiration risk (11). 

The resulting physiological deficits include reduced laryngeal excursion, BOT dysfunction, and reduced pharyngeal contractions. Notably, higher radiation doses to the pharyngeal constrictor muscles and supraglottic larynx have been associated with worse swallowing function and increased aspiration (10). Given the complex interplay among these factors, objective and quantitative assessments of swallowing function are crucial. Videofluoroscopic swallowing studies (VFSS) are considered the gold standard for diagnosing swallowing disorders and provide a detailed radiographic assessment of the structures and dynamics involved in all phases of the swallowing process. 

However, there remains a need for comprehensive analyses utilizing quantitative biomechanical indices to precisely identify predictive factors for swallowing outcomes and effectively guide personalized rehabilitation strategies (12). Accordingly, we aimed to comprehensively analyze the postoperative swallowing function in patients with oral and oropharyngeal cancer using VFSS-derived quantitative biomechanical indices as well as to identify independent predictors of key swallowing outcomes. 

## Materials and Methods

### Study Design and Participants

This cross-sectional study was conducted at the Department of Otolaryngology-Head and Neck Surgery, University Medical Center, Ho Chi Minh City (UMC), Vietnam, from October 2022 to March 2025. The UMC is a prominent state-owned public teaching hospital affiliated with the University of Medicine and Pharmacy in Ho Chi Minh City Vietnam. This study enrolled 60 patients diagnosed with previously untreated, pathologically confirmed squamous cell carcinoma at various oral and oropharyngeal sites, including the tongue, floor of the mouth, tonsils, soft palate, and pharyngeal wall. Participants were required to be aged ≥18 years, alert, cooperative, and have undergone the indicated surgery at the UMC. We excluded patients who had prior head and neck cancer at a different site, preoperative head and neck radiation therapy, pre-existing recurrent laryngeal nerve palsy, or a diagnosis of neuromuscular disorders. We included all eligible consecutive patients encountered during the study period without formal sample size calculations in order to ensure a diverse and representative patient population. Ethical approval was obtained from the Ethics Committee of the University of Medicine and Pharmacy at Ho Chi Minh City (approval number: 727/HĐĐĐ-ĐHYD). All the patients provided informed written consent.

### Sample Size Justification

The sample size was determined using a pragmatic rule-of-thumb for multivariate regression analysis. We applied generalized linear models (GLMs) to identify independent predictors, with the final models including up to five to six independent variables (e.g., sex, adjuvant treatment, T-stage, and suprahyoid defect). Accordingly, the enrollment of 60 consecutive patients was justified, which adhered to the widely accepted requirement of 10 to 15 participants per predictor in the model.

### Patient and Treatment Characteristics

Comprehensive preoperative evaluations included demographic data (age, sex, and body mass index), comorbidities, primary tumor location, and clinical tumor staging. The included patients underwent surgical tumor resection, which included unilateral or bilateral neck dissection where indicated, and defect reconstruction as necessary. 

A suprahyoid muscle defect was indicated by surgical cutting of the mylohyoid and geniohyoid muscles with or without the digastric and stylohyoid muscles (9). Furthermore, we recorded the postoperative adjuvant treatment status (radiotherapy or chemoradiotherapy). 

### Postoperative Swallowing Assessment

For patients with and without adjuvant therapy, VFSS was performed at 6–7 and 3 postoperative months, respectively. This former timing was selected to allow resolution of acute side effects such as mucositis (5). 

This timeframe is consistent with previous studies assessing post-treatment swallowing outcomes (6,8,10,12-14). The VFSS comprised measures of swallowing safety, efficiency, and key biomechanical parameters. VFSS were performed at the Department of Radiology using a Siemens Luminos Fusion radiographic fluoroscopy system. Digital recordings were made at 30 frames per second (fps) to facilitate slow-motion playback and analyses. 

During the procedure, patients were placed in an examination chair with a fluoroscopy unit in the lateral position. The patient's head was neutral and facing forward. 

**Fig 1:**
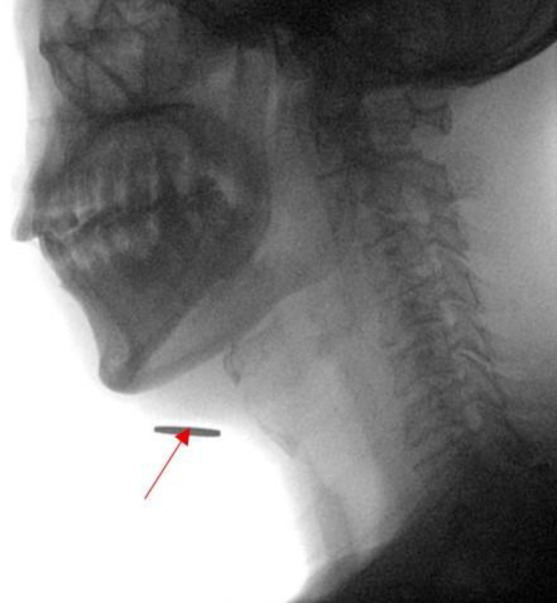
Boundaries of the lateral fluoroscopic view. A radiopaque disk is secured to the patient’s chin with tape to allow for later calibration (red arrow)

A 20-mm radiopaque disk secured to the patient’s chin with tape to allow for later calibration during the on-screen calculation of objective fluoroscopic swallowing measures. The boundaries of the fluoroscopic field in the lateral view were the lips anteriorly, the nasopharynx superiorly, the cervical spine posteriorly, and the cervical esophagus inferiorly (Figure 1). 

Patient radiation exposure was minimized using standard gonadal lead shielding, strictly adhering to the ALARA principle (As Low as Reasonably Achievable). The fluoroscopy time for each patient was <3 min. The protocol involved sequential assessment using different liquid consistencies and volumes as follows: 5 mL of moderately thick liquid, progressing to 10 mL, and then 20 mL if no aspiration occurred at the preceding volume. Additionally, we performed evaluations using 5 mL each of thin and extremely thick liquids. 

The liquid consistencies used for assessment were standardized based on the International Dysphagia Diet Standardization Initiative (IDDSI) framework: thin liquid (IDDSI Level 0), moderately thick liquid (IDDSI Level 3), and extremely thick liquid (IDDSI Level 4). A teaspoon was used to administer all 5 mL boluses, while a cup was used for the 10 mL and 20 mL boluses. 

These bolus types have been used in clinical protocols designed to evaluate patients’ responses to foods with different textures (3,15,16). 

### Assessment of Swallowing Safety and Efficiency

**Fig 2:**
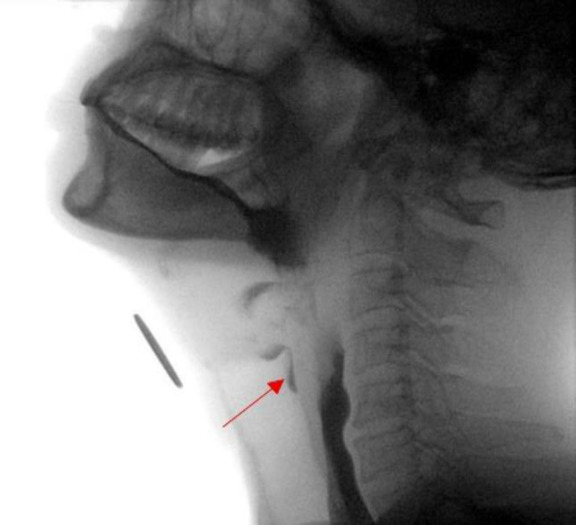
Aspiration with PAS scores = 8 (red arrow)

Swallowing safety was assessed for each bolus trial using an 8-point Penetration Aspiration Scale (PAS) (17). A PAS score of 1 indicated no airway invasion, scores of 2–5 indicated penetration (material above or contacting vocal folds), and scores of 6–8 indicated aspiration (material below the vocal folds) (18) (Figure 2). 

**Fig 3:**
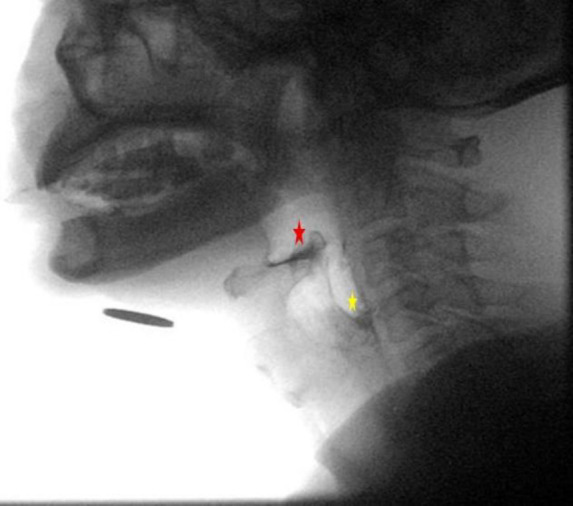
Post-swallow residue level 1 in the oropharynx (red asterisk) and hypopharynx (yellow asterisk)

The highest score was recorded for each bolus type. The swallowing efficiency (post-swallow residue) in the oropharynx (vallecula) and hypopharyngeal (piriform sinus) regions was quantified using an ordinal scale: 0 (no residue), 1 (trace residue), and 2 (significant residue) (19) (Figure 3).

### Quantitative VFSS measures

Time-based VFSS measurements were calculated by dividing the number of frames between two specific anatomical events by the recording rate (30 fps). The displacement measurements were obtained using the measuring tools. A single radiologist performed all VFSS measurements. 

**Fig 4:**
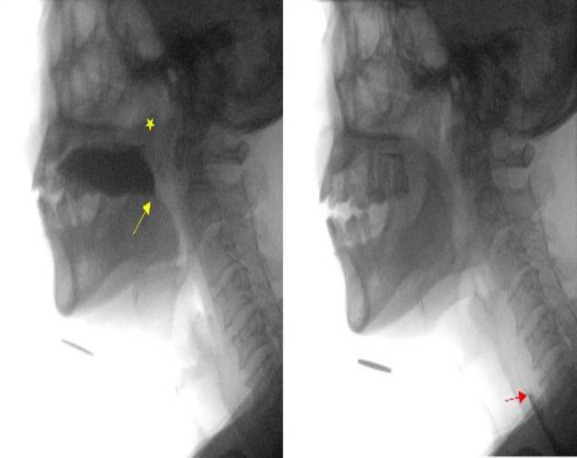
Lateral fluoroscopic views for determining the pharynageal transit time (PTT). PTT is calculated on PACS by identifying the frame where the bolus head (yellow arrow) has just passed the posterior nasal spine (yellow asterisk) (left panel) and the frame where the bolus tail has just passed the pharyngoesophageal segment (red arrow) (right panel). The time is determined by multiplying the number of frames between these two points by 1/30 (seconds)

**Fig 5:**
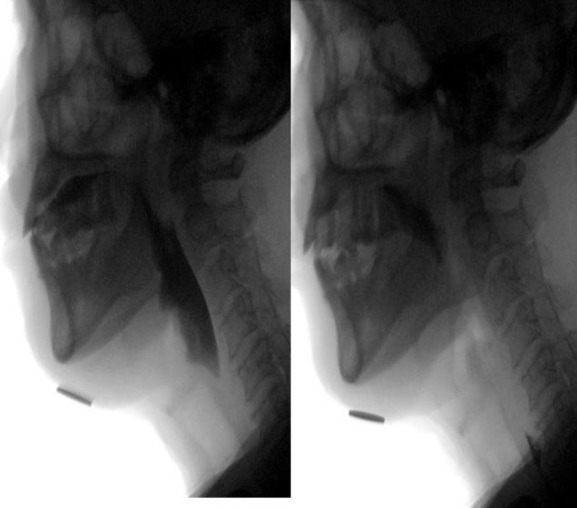
Laryngeal vestibule closure duration. On PACS, identify the frame where the arytenoid makes contact with the petiole of the epiglottis (left panel) and the frame where the epiglottis returns to its pre-swallow position (right panel). The laryngeal vestibule closure duration is the number of frames between these two points multiplied by 1/30 (seconds)

**Fig 6:**
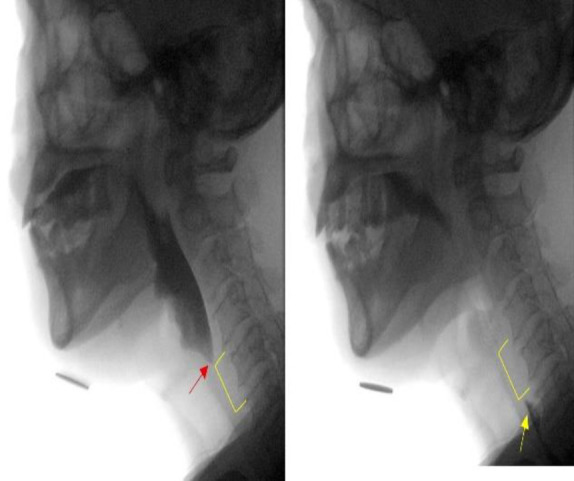
Pharyngoesophageal segment (PES) opening duration. On PACS, identify the frame where the bolus head (red arrow) first enters the PES (yellow bracket) (left panel) and the frame where the bolus tail (yellow arrow) leaves the PES (yellow bracket) (right panel). The PES opening duration is the number of frames between these two points multiplied by 1/30 (seconds)

**Fig 7:**
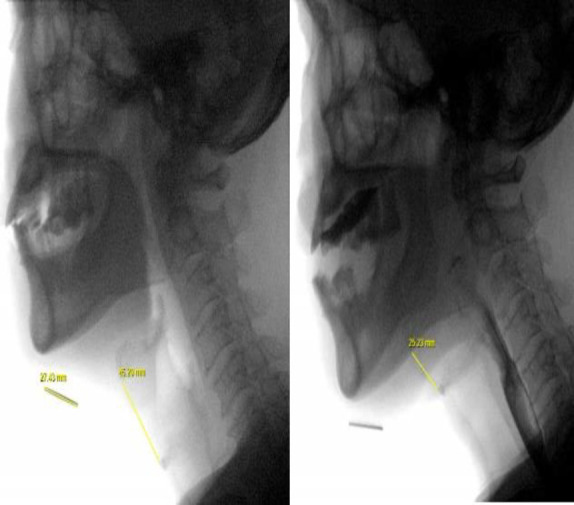
Measurement of maximum laryngeal elevation. The distance between the inferior border of the hyoid bone and the superior border of the cricoid cartilage at rest (45.20 mm) (left panel) is subtracted from the minimum distance during swallowing (25.23 mm) (right panel). The resulting value is then calibrated using the radiopaque disk (real diameter: 20 mm; measured diameter on PACS: 27.43 mm) to determine the true maximum laryngeal elevation (14.56 mm)

**Fig 8:**
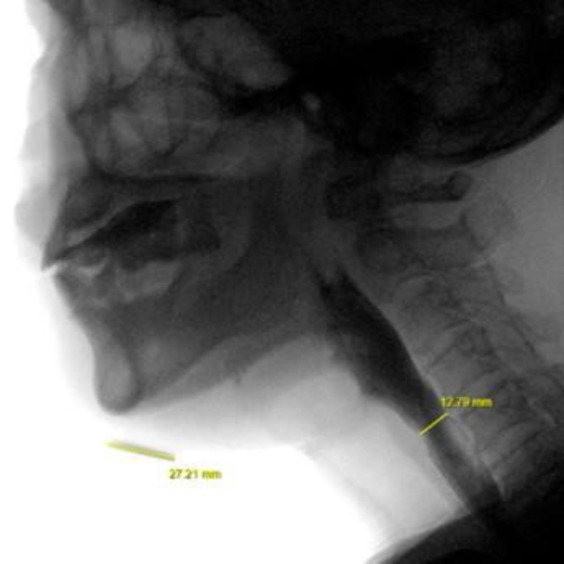
PES anterior-posterior opening. The narrowest point of opening between C3 and C6 in the lateral fluoroscopic view measured 12.79 mm on PACS (right yellow line). This value was calibrated using the radiopaque disk (left yellow line, 27.21 mm) to yield a true PES opening of 9.4 mm (12.79 x 20 / 27.21)

Specific measurements included (16):

- Pharyngeal transit time (PTT): time interval from the moment the bolus head passes the mandibular ramus until the bolus tail bolus passes through the PES (Figure 4).

- Laryngeal vestibule closure duration: the time interval from the arytenoid cartilage approximating the epiglottis petiole until the epiglottis returns to its pre-swallow resting position (Figure 5).

- PES opening duration: The time interval from the onset of pharyngoesophageal segment opening until the bolus closes (Figure 6).

- Maximum laryngeal elevation: relative distance between the hyoid and larynx at rest and at maximum approximation during swallowing (Figure 7).

- PES anteroposterior opening: The narrowest point of the opening between the pharynx and esophagus on the lateral fluoroscopic view, which was measured at the narrowest point between C3 and C6 (Figure 8).

### Data Analysis

Descriptive statistics (means ± standard deviation for continuous variables and frequencies and percentages for categorical variables) were used to summarize patient characteristics and outcomes.

 We performed GLM analysis to examine the relationships of swallowing biomechanical measures with various patient demographics, tumor characteristics, and treatment factors. Spearman’s correlation analysis was used to examine the association between clinical characteristics and categorized oropharyngeal residues. Statistical significance was set at p < 0.05. 

 All statistical analyses were performed using SPSS version 20.0 (IBM Corporation, Armonk, New York, United States).

## Results

### Clinical and demographic characteristics of patients

The clinical and demographic characteristics of the 60 patients are summarized in Table 1.

The study cohort (N=60) was predominantly male (78.3%), with a mean age and BMI of 54.28 ± 11.97 years and 22.41 ± 3.18, respectively. Tumors were most frequently located in the oral cavity (71.7%), specifically on the tongue and floor of the mouth (71.7%), whereas oropharyngeal sites accounted for 28.3% of cases. The most common oropharyngeal sites were the tonsils (20.0%). Most tumors were classified as T2 stage tumors (58.3%). Regarding treatment, 58.6% of patients did not undergo intraoperative neck dissection. Adjuvant therapy (radiotherapy or chemoradiotherapy) was administered to 58.4% of the patients. A suprahyoid muscle defect was present in 61.7% of the patients.

**Table 1:** Clinical and demographic characteristics of included patients

**Characteristics**	**N (%) or mean ± SD (Min-Max)**
Age (years)	54.28 ± 11.97 (26-77)
BMI (kg/m^2^)	22.41 ± 3.18 (16.5-28.8)
Sex	
Male	47 (78.3%)
Female	13 (21.7%)
Anatomical site of cancer	
Oral Cavity	43 (71.7%)
Tongue - floor of mouth	43 (71.7%)
Oropharynx	17 (28.3%)
Base of tongue	1 (1.7%)
Tonsil	12 (20.0%)
Soft palate	1 (1.7%)
Posterior pharyngeal wall	3 (5.0%)
Clinical Tumor Stage	
T1	4 (6.7%)
T2	35 (58.3%)
T3	12 (20.0%)
T4	9 (15.0%)
Neck dissection	
No	34 (58.6%)
Yes	24 (41.4%)
Suprahyoid muscle defect	
No	23 (38.3%)
Yes	37 (61.7%)
Adjuvant radiotherapy or chemoradiotherapy	
No	25 (41.7%)
Adjuvant radiotherapy	19 (31.7%)
Adjuvant chemoradiotherapy	16 (26.7%)

### Proportions of PAS score categories across 5mL liquid consistencies

Table 2 presents the frequency distribution of the categorized PAS scores for 5mL boluses across the liquid consistencies.

**Table 2:** Distribution of categorized PAS scores for 5mL liquid consistencies

**PAS Category** **(5mL Bolus)**	**No Penetration - Aspiration ** **(N, %)**	**Penetration** **(N, %)**	**Aspiration ** **(N, %)**	**N**
5 mL of moderately thick liquid	53 (93.0%)	2 (3.5%)	2 (3.5%)	57
5 mL of thin liquid	41 (70.7%)	10 (17.2%)	7 (12.1%)	58
5 mL of extremely thick liquid	59 (98.3%)	1 (1.7%)	0 (0.0%)	60

The distribution of penetration-aspiration events showed notable variances across the different liquid consistencies. For 5 mL of thin liquid, 17.2% and 12.1% of patients experienced penetration and aspiration, respectively. Contrastingly, for 5 mL of moderately thick liquid, the rates of penetration and aspiration were significantly lower (3.5% for each category). Notably, for 5 mL of extremely thick liquid, only one patient (1.7%) demonstrated penetration, and no aspiration was recorded.

### Multivariate analysis (GLM) for 20mL moderately thick liquid VFSS measures

GLMs were used to identify the independent predictors of VFFS measures with 20 mL of moderately thick liquid. 

Gamma distribution and log link function were used for these models (Table 3).

**Table 3:** Independent predictors of VFSS measures with 20 mL of moderately thick liquid (N = 60)

**Dependent VFSS measure (20mL moderately thick liquid)**	**Predictor**	**Coefficient (B)**	**p-value**	**Direction of Effect**
Laryngeal elevation	Sex (male vs. female)	0.456	0.016	Higher (for male)
	Suprahyoid muscle defect (no vs. yes)	0.559	0.001	Higher (for no defect)
Pharyngeal transit Time	Adjuvant treatment (no vs. yes)	-0.361	0.036	Shorter (for no adjuvant treatment)
PES opening duration	Tumor stage (early vs. advanced)	0.695	<0.001	Higher (for early stage)
	Suprahyoid muscle defect (no vs. yes)	-0.395	0.013	Shorter (for no defect)
	Age	0.016	0.012	Increases (with increasing age)
PES anterior -posterior opening	Adjuvant treatment (no vs. yes)	0.247	0.020	Higher (for no adjuvant treatment)
	Age	0.007	0.017	Increases (with increasing age)

Here, sex and suprahyoid muscle defects were independent predictors of laryngeal elevation. Adjuvant treatment was an independent predictor of pharyngeal transit times. Tumor stage, suprahyoid muscle defects, and age were independent predictors of the PES opening duration. Finally, adjuvant treatment and age were independent predictors of the PES anterior-posterior opening, while tumor stage independently predicted the lateral opening of the PES.

### Spearman correlation results for categorized oropharyngeal residue (20 mL of moderately thick liquid) (N=60)

We examined the associations between clinical characteristics and the categorized oropharyngeal residues using Spearman’s correlation analysis (Table 4).

**Table 4:** Spearman correlation results for categorized oropharyngeal residue (20 mL of moderately thick liquid**)**

**Relationship**	**Spearman's rho (r)**		**p-value (2-tailed)**	**Strength and direction**
Oropharyngeal residue * Tumor stage (advanced)		0.411	0.001	Moderately positive
Oropharyngeal residue * Neck dissection (yes)		0.391	0.002	Moderately positive
Oropharyngeal residue * Adjuvant treatment (yes)		0.345	0.008	Moderately positive
Gender * Categorized oropharyngeal residue		-0.060	0.655	Not significant
Anatomical site of cancer * Categorized oropharyngeal residue		-0.021	0.874	Not significant
Age group * Categorized oropharyngeal residue		-0.061	0.651	Not significant

Oropharyngeal residues showed a moderately positive correlation with advanced tumor stage, neck dissection, and adjuvant treatment. Conversely, there was no significant correlation of oropharyngeal residues with sex, anatomical site of cancer, or age. Additionally, there was no significant association between hypopharyngeal residue and advanced tumor stage. 

## Discussion

This study identified several clinical and demographic factors that could independently predict swallowing outcomes, offering crucial insights for optimizing patient management and tailoring rehabilitation strategies. Within the broader scientific context, the assessment of dysphagia in patients with head and neck cancer (HNC) has shifted from subjective patient-reported outcomes to objective quantitative measures (4). 

Subjective patient-reported outcomes and general swallowing safety scales are valuable; however, they often fail to capture the underlying physiological deficits, which is essential for developing effective and personalized interventions (2). By focusing on quantitative metrics, including pharyngeal transit time, laryngeal elevation, and PES opening, this study addresses a critical gap in the literature by allowing further elucidation of the biomechanical mechanisms underlying post-treatment dysphagia (4,20).

### Postoperative airway protection and bolus consistencies

There was a significant incidence of postoperative penetration and aspiration in patients with HNC, especially with thin liquids. Thin liquids yielded the highest rates of penetration (17.2%) and aspiration (12.1%), while extremely thick liquids yielded considerably lower rates (1.7% penetration and 0% aspiration). Normally, a thin liquid bolus moves quickly through the pharynx, which requires rapid and well-coordinated airway protection mechanisms, including laryngeal elevation and vocal fold adduction to prevent material from entering the trachea. Conversely, thick liquids have a significantly reduced speed of bolus flow, which provides more time for compromised airway protection mechanisms to function effectively (15). In postoperative patients with HNC, these protective mechanisms are often compromised by tissue resection, cranial nerve damage, or post-radiotherapy fibrosis. Delayed swallow reflex or reduced laryngeal elevation can create a critical time window for a fast-moving thin liquid bolus to spill into the airway, which leads to penetration or aspiration (21). This is consistent with previous reports of high frequencies of aspiration within the immediate to 6-month post-treatment period, with frequencies of ≈28.6% at <3 post-treatment months; moreover, higher aspiration rates have been observed with thin liquids than with thick liquids, including puddings or biscuits (3). Another study reported abnormal PAS scores in 45% of post-treatment swallowing studies, which highlights the commonality of airway infiltration (22). There remains no specific evidence regarding the efficacy of thickened liquids in the HNC population (23). 

However, this study provided observational evidence regarding their utility as a safe and effective compensatory strategy for minimizing the risk of aspiration, which is a major cause of pneumonia and mortality (21).

### Independent Predictors of Laryngeal Elevation: Focus on Suprahyoid Integrity

Our GLM analysis identified suprahyoid muscle defects as the most significant independent predictor of reduced laryngeal elevation, with sex being another predictor. The suprahyoid muscles, including the geniohyoid and mylohyoid muscles, are crucial for hyoid bone movement, which is essential for airway protection and efficient bolus passage into the esophagus (15). Damage to these muscles, including following extensive resections for oral cancer, is associated with significantly reduced hyoid bone movement, which is linked to severe postoperative swallowing dysfunction (7,9). This demonstrates that the extent of surgical resection is a core determinant of subsequent biomechanical deficits. Insufficient laryngeal elevation directly compromises airway protection and reduces the PES opening, which leads to an increased risk of post-swallowing residue and aspiration risk (15). 

This finding highlights the importance of implementing targeted, high-intensity exercises focused on strengthening the remaining suprahyoid muscles (e.g., the Mendelsohn maneuver, effortful swallows, or head-lift exercises). The observed higher laryngeal elevation in males is consistent with previously reported sex differences in hyolaryngeal motion (20,21). Nonetheless, the actionable clinical priority is intervening against surgery-related defects to maximize residual function.

### Adjuvant Treatment as an Independent Predictor for Prolonged Pharyngeal Transit Time

Adjuvant treatment is an independent predictor of prolonged PTT, which is a crucial clinical marker of impaired pharyngeal contraction and reduced propulsive force, typically secondary to radiation-induced tissue fibrosis and muscle atrophy (23-26). Prolonged PTT causes pharyngeal stasis, which indicates that the bolus remains in the pharynx for an extended period. The clinical significance of prolonged PTT was strongly corroborated by the moderately positive correlation between adjuvant treatment and oropharyngeal residues (Table 4). This dual deficit (impaired timing and high residue levels) results in insufficient clearance and an increased risk of secondary aspiration. Therefore, patients receiving adjuvant therapy should be prioritized for rehabilitation programs that emphasize the restoration of propulsive power and pharyngeal clearance, utilizing techniques such as the effective swallow maneuver to actively reduce PTT and mitigate the long-term risk of aspiration (27).

### Multi-factorial Determinants of Pharyngoesophageal Segment Dysfunction

In our study, tumor stage, suprahyoid muscle defect, adjuvant treatment, and age were independent predictors of PES function. Specifically, we observed two primary mechanisms underlying PES dysfunction. First, regarding the impairment of timing and efficacy, the PES opening duration was significantly shorter for advanced tumor stages. 

This can be attributed to a severe and independent failure of the system to maintain the necessary clearance window owing to the profound structural damage caused by the advanced disease. Conversely, the presence of a suprahyoid defect was associated with a prolonged PES opening duration, which can be attributed to motor inefficiency and compensatory struggle. Furthermore, duration showed a negative and positive correlation with functional efficiency and age, respectively, which reflects general systemic motor weakness. Second, regarding the compromised physical dimension and distensibility, the PES anteroposterior opening (dimension) was independently and significantly reduced by adjuvant treatment. However, in our model, this dimension was positively correlated with age. This finding is inconsistent with established evidence regarding presbyphagia, which dictates that physiological aging compromises the distensibility of the pharyngeal and UES muscles, leading to reduced dimensions (28). Our findings may reflect a type I error associated with the small sample size (N=60) or the presence of uncontrolled confounding factors specific to this highly selected surgical cohort. On the other hand, adjuvant radiotherapy contributes to tissue fibrosis and rigidity, which directly hinders maximal PES (23-26, 29). Taken together, the determinants of PES dysfunction fall into two distinct categories. The first category comprises factors related to primary disease and resection (tumor stage and suprahyoid defect), which impair the complex biomechanical timing and efficacy of the opening. The second category comprises systemic factors (adjuvant treatment and age), which compromise the physical dimensions and distensibility of the segment. Clinically, this comprehensive profile is a valuable risk stratification tool. Here, patients with advanced tumor stages and older patients experience the most severe PES deficits, which are characterized by poor timing and restricted size. Accordingly, there is a need for proactive management, potentially involving specialized techniques, including balloon dilation or botulinum toxin injection (in case of suspected cricopharyngeal dysfunction), in addition to intensive swallowing therapy to ensure functional PES opening (30,31).

### Clinical Significance of Predictors of Oropharyngeal Residue

The presence of post-swallow residues, particularly in the oropharynx, is a crucial objective indicator of poor bolus propulsion and impaired pharyngeal clearance (9,30,32). We found that oropharyngeal residues were moderately correlated with adjuvant treatment, advanced tumor stage, and neck dissection (Table 4). Although this correlation is associated with functional timing deficits, the correlation with advanced tumor stage and neck dissection is highly significant for patient risk stratification. This suggests that the surgical extent and disease burden are primary drivers of swallowing inefficiency. For clinical management, patients with advanced T-stage disease or those undergoing neck dissection should be classified as having the highest risk of chronic residue and potential late-onset aspiration (1). 

Accordingly, there is a need for proactive surveillance using VFSS as well as early prescription of robust compensatory and strengthening strategies (including sequential swallowing or the effortful swallow maneuver) to ensure effective pharyngeal clearance (10,11). Although hypopharyngeal residues was not significantly associated with advanced tumor stage, the strong correlation with oropharyngeal residues emphasizes the importance of targeting these specific deficits.

### Limitations and future directions

This study has several limitations. First, it had a cross-sectional design and did not collect preoperative (baseline) VFSS data. This impeded precise quantification of functional differences attributable solely to treatment and precludes the establishment of a direct causal relationship. Therefore, our findings should be interpreted as identifying associations and independent predictors rather than causation. Second, all the VFSS biomechanical measurements were performed by a single trained radiologist. Although this ensures high internal consistency (minimizes inter-rater variability), it may introduce single-observer bias. Third, the cohort was disproportionately male (78.3%).

 This is consistent with previously reported sex differences in hyolaryngeal motion (21,22); nonetheless, it necessitates caution in interpreting sex-specific predictors. Future studies should aim for a more balanced cohort. Finally, given the small sample size (n=60), further large-scale multi-center cohort studies are warranted to validate these independent predictors.

## Conclusion

In conclusion, our study reinforces that postoperative dysphagia in patients with HNC is a complex issue that is profoundly influenced by the surgical extent, adjuvant therapy, and tumor characteristics. VFSS analysis could highlight specific biomechanical deficits; further, we identified predictors of key swallowing outcomes, including sex, adjuvant treatment, and suprahyoid defects. 

These findings highlight the need for precise objective assessments using VFSS to guide personalized rehabilitation efforts. The observed low rates of aspiration with extremely thick liquids suggest that modifying liquid consistency is a safe and effective compensatory strategy to minimize aspiration risk in this patient population.
